# Supplementary material for: Epidemic modelling of monitoring public behavior using surveys during pandemic-induced lockdowns
Source: Commun Med (Lond). 2023 Jun 8;3:80. doi: 10.1038/s43856-023-00310-z (PMC10249934; doi:10.1038/s43856-023-00310-z)
Supplement: Supplementary file 1 — Description of Additional Supplementary Files [file 43856_2023_310_MOESM1_ESM.pdf]

Description of Additional Supplementary File

File Name: Supplementary Data

Description: The regional hospitalization data together with the predictors used in the main text
