# Supplementary material for: Epidemic modelling of monitoring public behavior using surveys during pandemic-induced lockdowns
Source: Commun Med (Lond). 2023 Jun 8;3:80. doi: 10.1038/s43856-023-00310-z (PMC10249934; doi:10.1038/s43856-023-00310-z)
Supplement: Supplementary file 2 — Supplementary Information [file 43856_2023_310_MOESM2_ESM.pdf]

Epidemic modelling of monitoring public behavior using surveys during pandemic-induced lockdowns

Andreas Koher, Frederik Jørgensen, Michael Bang Petersen, Sune Lehmann

Supplementary Note 1 - Extended data discussion: Survey

We contact participants via eBoks, the official electronic mail system of public authorities, and provide no financial incentives. Notably, about 8 % of the Danish population, mainly older people, are exempted from eBoks. Despite this limitation and a response rate of 25 %, the participants are representative of the broad Danish population regarding the stratified characteristics [1]. Further details on sampling and questions are available in [1].

From our survey, we select the self-reported number of contacts within a two-meter distance for at least 15 minutes and differentiate between contacts to family members, friends, colleagues and strangers, where the latter refers to all remaining contact types. Our analysis focuses on the period from 2020-12-01 to 2021-02-01 with 15.595 participants split into the five regions of Denmark: Region Hovedstaden (Capital), Region Midtjylland (Center), Region Nordjylland (North), Region Sjælland (Zealand), Region Syddanmark (South). We remove unreasonable outliers that include negative numbers and values above 50, 100, 100 and 1000 for contacts to family members, friends, colleagues and strangers, respectively, thus dropping 0.3% of all responses. By summing up the reported number of context-dependent contacts, we obtain every survey participant's total number of contacts. Next, we mark a participant as risk-taking, either context-depending or in terms of total contacts, and derive the daily fraction of risk-takers. This quantity is robust to outliers and reflects our understanding that super-spreading events drive Covid-19 infections [2]. Finally, we take a 7-day moving average, centered on day four and calculate the change in behaviour relative to the first observation day, i.e., 2020-12-01. We thus have five data streams for each of the five regions of Denmark: risk-taking behaviour given overall contacts and four context-depending time series (see Fig. 1 panel B in the main text and Supplementary Fig. 6), respectively.

Supplementary Note 2 - Extended data discussion: Mobility

Apple provides three data streams, namely, *driving*, *walking*, and *transit*. The latter is not available in all regions of Denmark, and therefore we exclude it from the analysis.

The Google data includes six time-series: *grocery & pharmacy*, *retail & recreation*, *transit stations*, *workplaces*, *parks*, and *residential*. We exclude *parks* because data is too sparse on a regional level. In addition to the individual data streams, we combine *driving* and *walking* to a single *Apple* time series, and equally for *Google*, we use *grocery & pharmacy*, *retail & recreation*, *transit stations*, and *workplaces* as suggested in [3].

The telecommunication (telco) time series derives from aggregated mobility flows within and between municipalities. Denmark's leading mobile network operators provided the data to the Statens Serum Institut (SSI), covering 2020-02-01 to 2021-06-30. The SSI officially requested the data to improve national Covid-19 models and understand population behaviour in response to non-pharmaceutical interventions. Detailed information on the data is available in [4] and the complete data set can be downloaded from [5].

As a final preprocessing step to the mobility data from Apple, Google and the telco companies, we take a 7-days moving average and calculate the change in mobility relative to the first observation day on 01-December-2020.

Supplementary Note 3 - MCMC sampling

We implement the epidemiological model in the Julia programming language [6] using the Turing.jl package [7] for Bayesian inference. In particular, we use the No-U-Turn sampler [8], i.e. a variant of the Hamilton Monte-Carlo sampler with a target acceptance rate of 0.99 and a maximum tree-depth of 8. We draw 5000 samples from 5 chains each and discard the first 1000 for warm-up.

All inference results report no divergent transitions. Also, the maximum Gelman–Rubin diagnostic and $\hat{R}$ statistics is below 1.1 for all simulations, thus indicating sufficient mixing and convergence of the Monte-Carlo chains.

Further implementation details and a step-by-step tutorial to reproduce the main results are available on GitHub [9].

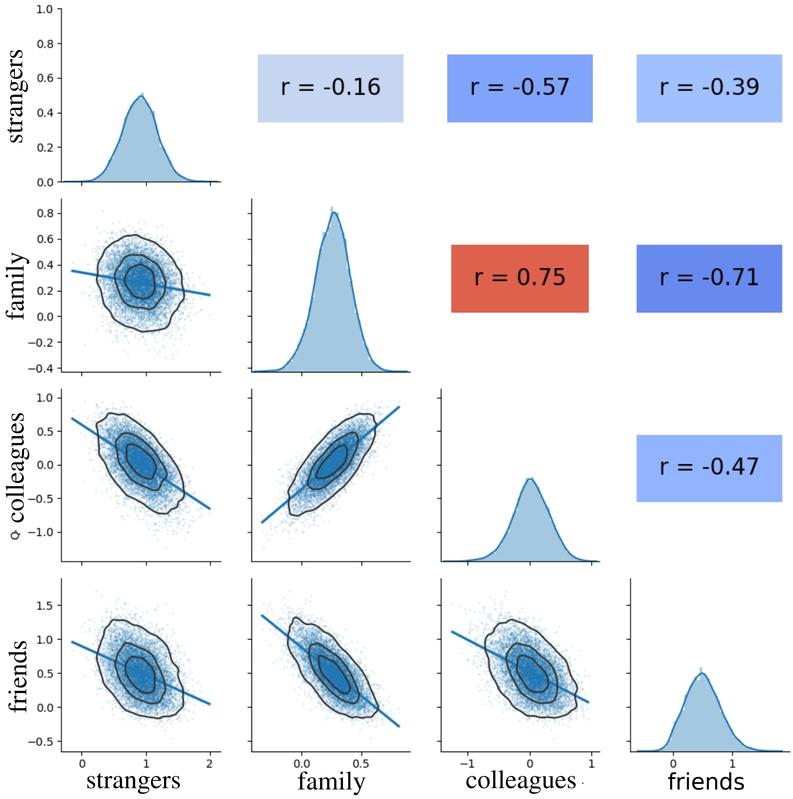

Supplementary Figure 1 Positive cross-correlation suggests a combination of risk-taking behaviour towards colleagues and family members for monitoring. The details: We compare risk-taking behaviour with a threshold at the 70th percentile towards different social groups, i.e., contacts to strangers, family members outside the household, friends, and colleagues. The diagonal shows pooled posterior effect sizes. The upper non-diagonal fields give the Pearson's correlation coefficient, whereas the lower non-diagonal fields present more details: a scatter plot of sampled effect sizes from two different predictors with contours of constant density and a linear regression line, which visualizes the correlation. The figure shows that pooled effect sizes for risk-taking behaviour towards strangers, friends, and colleagues are negatively correlated indicating co-linearity of the corresponding time-series (see Supplementary Fig. 6 for a visual comparison). Note that family and colleagues related effect sizes are positively correlated, thus suggesting a combination of both time series. Indeed, we find that risk-taking behaviour towards colleagues and family members together have a similar predictive performance to the best model (see Supplementary Table 1.

rank	predictor	score difference	score difference (std)
**0**	**friends**	**-0.0**	**0.0**
**1**	**family & colleagues**	**-3.85455**	**4.91707**
**2**	**strangers**	**-12.3089**	**7.52822**

Supplementary Table 1 A combination of risk-taking behaviour towards colleagues and family members shows a comparable predictive performance as the best model. In detail, we define risk-taking behaviour with a threshold at the 70th percentile, calculate the PSIS-LOO score, which approximates the out-of-sample predictive performance, and rank the results from highest to lowest performing. We consider the score difference significant if it is larger than the 95% CI (approx. twice the standard error) and highlight rows with bold letters that show a non-significant performance difference to the best performing model. Here, the difference in LOO score is non-significant for all model.

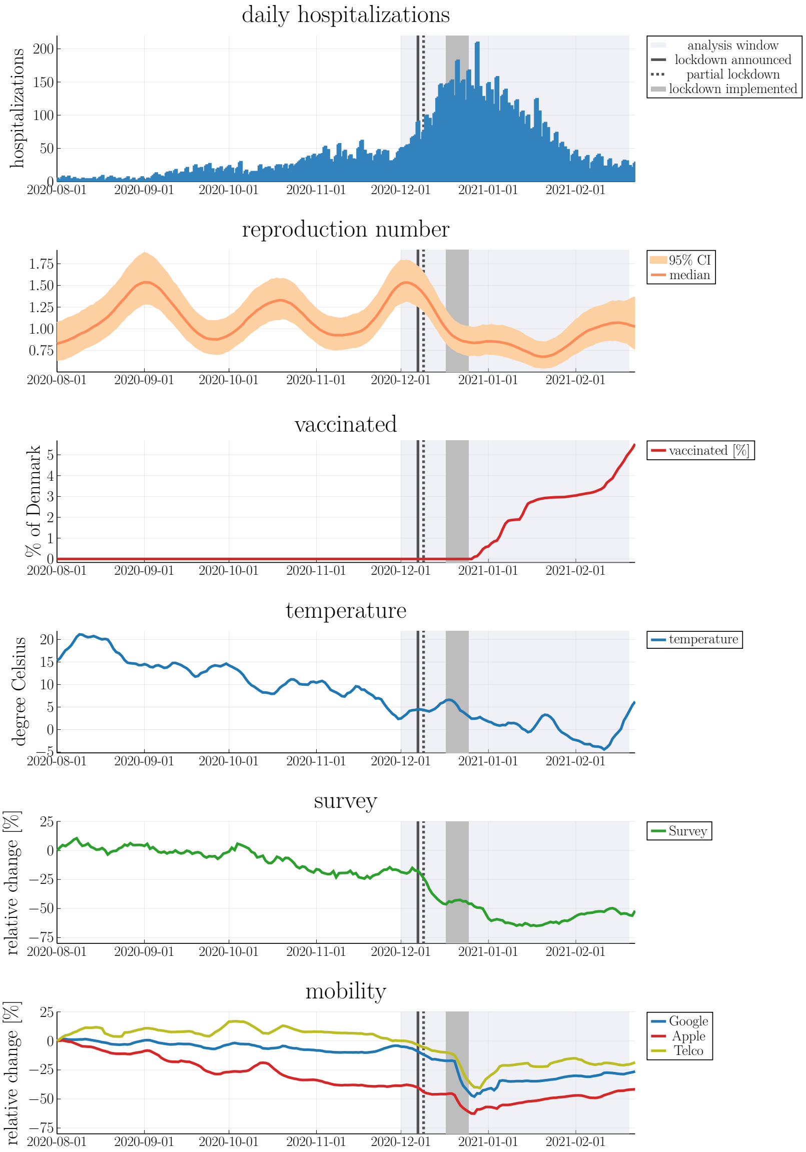

Supplementary Figure 2 top panel: national hospitalizations from 2020-08-01 to 2021-02-20. We highlight the analysis window from 2020-12-01 to 2021-02-01, which is the focus of our paper, the lockdown's announcement, it's partial and nation-wide implementation. 2nd panel: inferred reproduction number R_t from national hospitalizations. 3rd panel: fraction of Denmark's vaccinated population. 4th panel: average daily temperature in Denmark. 5th panel: Risk-taking behaviour with a threshold at the 70th percentile. 6th panel: Aggregated mobility data from Google, Apple and telecommunication providers (*Telco*). The visual comparison between reproduction number and predictors, including our survey and moblity, demonstrates the limitations: All predictors decrease significantly in correspondance to the lockdown, however, neither mobility nor our survey correlate well with the reproduction number during the early phase. There are potentially many limiting factors, including vaccination campaigns (3rd panel), changing masking efforts, and seasonal effects (4th panel). To improve future surveys and potentially predict the onset of the second wave, it would be relevant to know whether contacts occurred inside or outside, especially as temperatures drop and individuals adjust their behaviour.

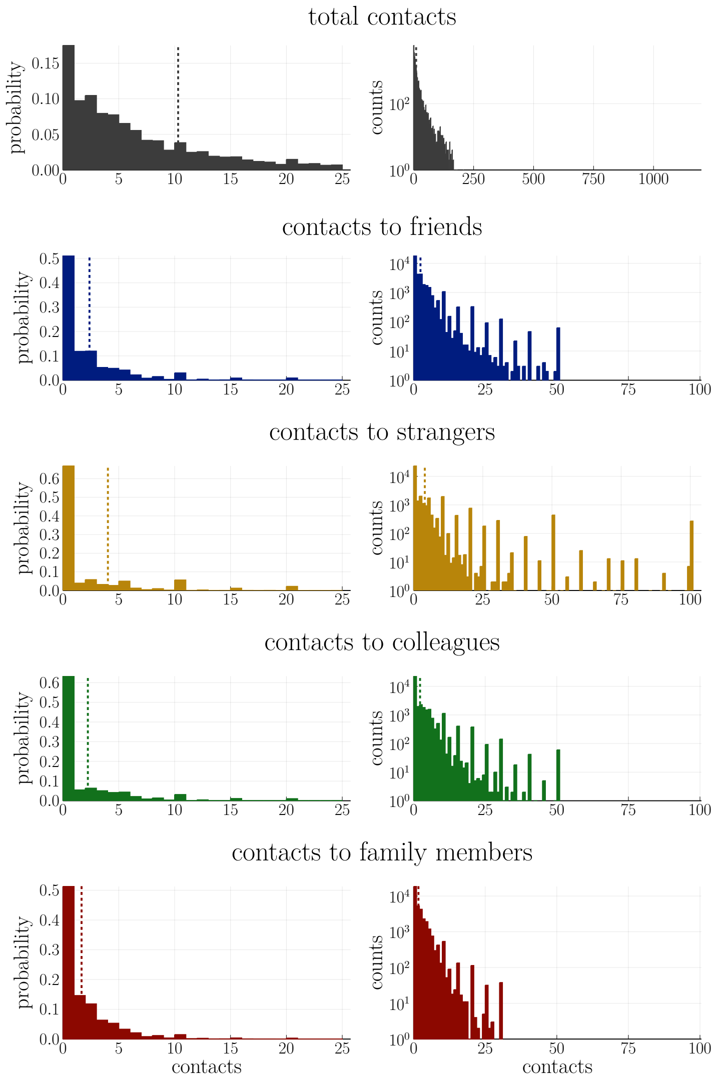

Supplementary Figure 3 Histogram over the reported contacts from 2020-08-01 to 2020-12-01. Left column: linear scaling with normalization to probabilities. Right column: Log scale without normalization, i.e. bins represent the number of survey participants that reported the corresponding number of close contacts. The linear scaling highlights the large fraction of individuals that report zero close contacts in the past 24h, whereas the log-scaling demonstrates the broad distribution of contacts, even after removing outliers as described in Supplementary Note 1. We use these statistics to define risk-taking behaviour in the main text as follows: Given a threshold in terms of a percentile, we derive the corresponding number of contacts from the above distributions. Then, we mark individuals as risk-taking (towards the total number of contacts or context-dependent) if they report more than the threshold number of contacts and report the daily fraction of risk-taking individuals. The resulting time-series captures subtle behavioural changes in the population and is robust with respect to outliers.

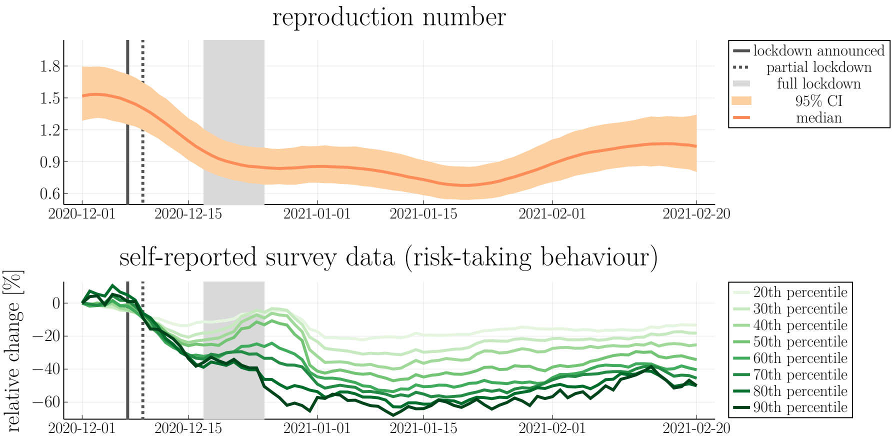

Supplementary Figure 4 Comparison between R_t and risk-taking behaviour. Top panel: reproduction number R_t, derived from national hospitalizations. Lower panel: changes in risk-taking behaviour given the total number of contacts and different thresholds in terms of percentiles (see Supplementary Fig. 3 for details). Similar to Fig. 1 in the main text, but includes more thresholds. Risk-taking behaviour that is derived from a larger threshold, shows an increased response to the lockdown's announcement and a smaller Christmas-related peak. Visually, this dynamics corresponds well to R_t and we confirm the improved predictive performance quantitatively in Supplementary Table 2 using the PSIS-LOO score.

**rank**	**contacts**	**percentile**	**score difference**	**score difference (std)**
**0**	> 6	**60th**	**-0.0**	**0.0**
**1**	> 9	**70th**	**-0.696663**	**2.36629**
**2**	> 7	**65th**	**-2.1874**	**1.5744**
**3**	> 11	**75th**	**-2.92527**	**3.75475**
**4**	> 18	**85th**	**-4.25635**	**5.00422**
**5**	> 14	**80th**	**-4.97029**	**5.52294**
6	> 5	55th	-5.98605	2.38119
**7**	> 24	**90th**	**-11.7012**	**6.85762**
8	> 4	50th	-13.6783	4.1538
9	> 2	40th	-16.0057	3.7325
10	> 3	45th	-21.8684	4.5585
11	> 1	30th	-22.1058	4.41053
12	> 0	20th	-28.049	5.57477

Supplementary Table 2 Predictive performance for different definitions of risk-taking behaviour. In detail, we compare thresholds that define risk-taking behaviour given the total number of contacts. As a threshold, we use the percentile of all reported contacts before the lockdown's announcement, i.e. from 2020-08-01 to 2020-12-01 (see first panel in Supplementary Fig. 3), and provide the corresponding number of contacts in a separate column. We calculate the PSIS-LOO score [10], which approximates the out-of-sample predictive performance and rank the results from highest to lowest performing. We consider the score difference significant if it is larger than the 95% CI (approx. twice the standard error) and highlight rows with bold letters that show a non-significant performance difference to the best performing model. Here, the 60th percentile performs best but, all thresholds above the 55th percentile are only insignificantly worse.

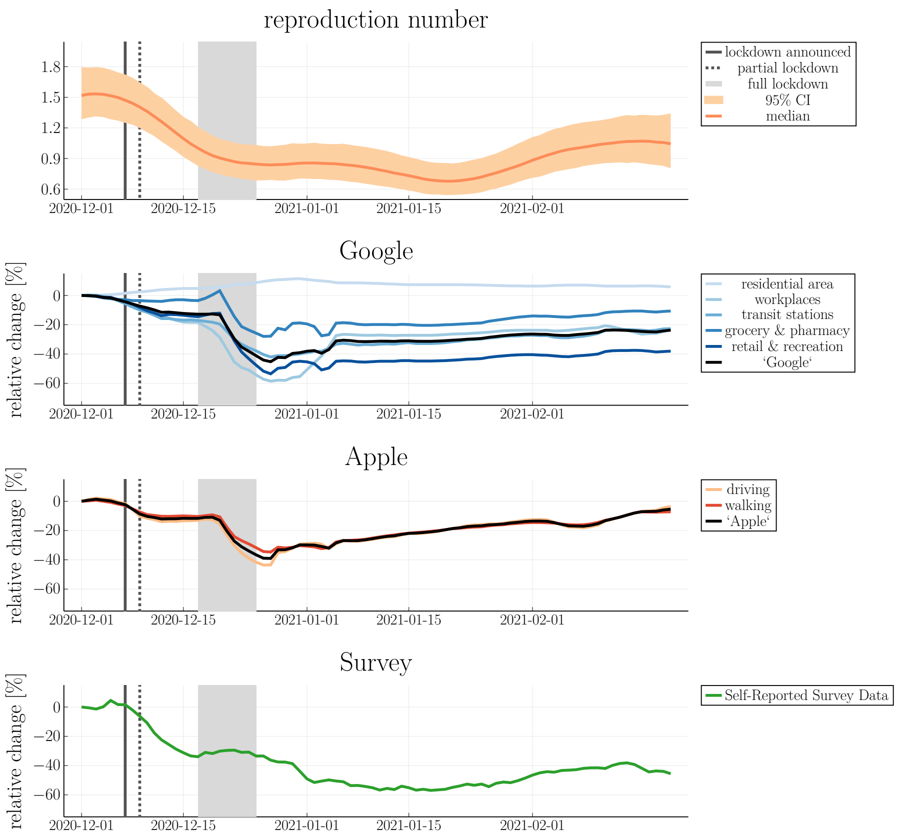

Supplementary Figure 5 National-level comparison between R_t and individual data streams from Google and Apple. 1st row: inferred reproduction number from national hospitalizations. 2nd row: Individual data streams from Google mobility trends [11]. We excluded the time-series "parks" because of too many missing values on the regional level. The combined time-series *Google* [3] includes *transit station*, *workplaces*, *retail & recreation*, *grocery & pharmacy*. 3rd row: Individual data streams from Apple mobility trends [12]. We exclude *transit* because of too many missing values on the regional level. The combined time series *Apple* [3] both remaining data streams. The comparison reveils that individual data streams from Google vary substantially whereas *driving* and *walking* from Apple show a similar dynamics. In Supplementary Table 3, we compare the predictive performance of individual mobility data streams with risk-taking behaviour from our survey (3rd row).

rank	predictor	score difference	score difference (std)
**0**	**Survey**	**-0.0**	**0.0**
**1**	**Google (retail & recreation)**	**-4.14564**	**4.92608**
2	Google (grocery & pharmacy)	-15.8254	7.32822
3	Google	-23.2743	7.95421
4	Google (transit stations)	-23.4502	7.42688
5	Apple (driving)	-92.5306	12.6706
6	Apple	-116.769	13.3046
7	Google (workplaces)	-130.899	13.1453
8	Apple (walking)	-138.908	13.4934

Supplementary Table 3 Self-reported survey data (*Survey*) demonstrates highest predictive performance compared to individual data streams from Google and Apple mobility, though, the performance difference to Google's *retail & recreation* is non-significant. The details: Survey data refers to risk-taking behaviour on the total number of contacts with a threshold at the 70th percentile. We calculate the PSIS-LOO score, which approximates the out-of-sample predictive performance and rank the results from highest to lowest performing. We consider the score difference significant if it is larger than the 95% CI (approx. twice the standard error) and highlight rows with bold letters that show a non-significant performance difference to the best performing model. See Supplementary Fig. 5 for a visual comparison of the time-series data. Interestingly, the score difference to Google's *retail & recreation* is non-significant. This observation appears plausible given (a) the increased risk for super-spreading events in retail and recreation spaces and (b) our results on risk-taking behaviour in different social contexts: Risk-taking behaviour towards friends and strangers predict hospitalizations best (Supplementary Table 5) and the latter correlates well with Google's *retail & recreation* (see Supplementary Fig. 9).

rank	predictor	score difference	score difference (std)
**0**	**survey**	**-0.0**	**0.0**
1	google	-23.3503	7.98878
2	telco	-69.3159	11.3415
3	apple	-116.649	13.3013

Supplementary Table 4 Self-reported survey data (*Survey*) demonstrates highest predictive performance compared to Google mobility, Apple mobility and telecommunication data (*Telco*). The details: Survey data refers to risk-taking behaviour on the total number of contacts with a threshold at the 70th percentile. We calculate the PSIS-LOO score, which approximates the out-of-sample predictive performance and rank the results from highest to lowest performing. We consider the score difference significant if it is larger than the 95% CI (approx. twice the standard error) and highlight rows with bold letters that show a non-significant performance difference. See Fig. 1 from the main text and Supplementary Fig. 5 for a visual comparison of the time-series data.

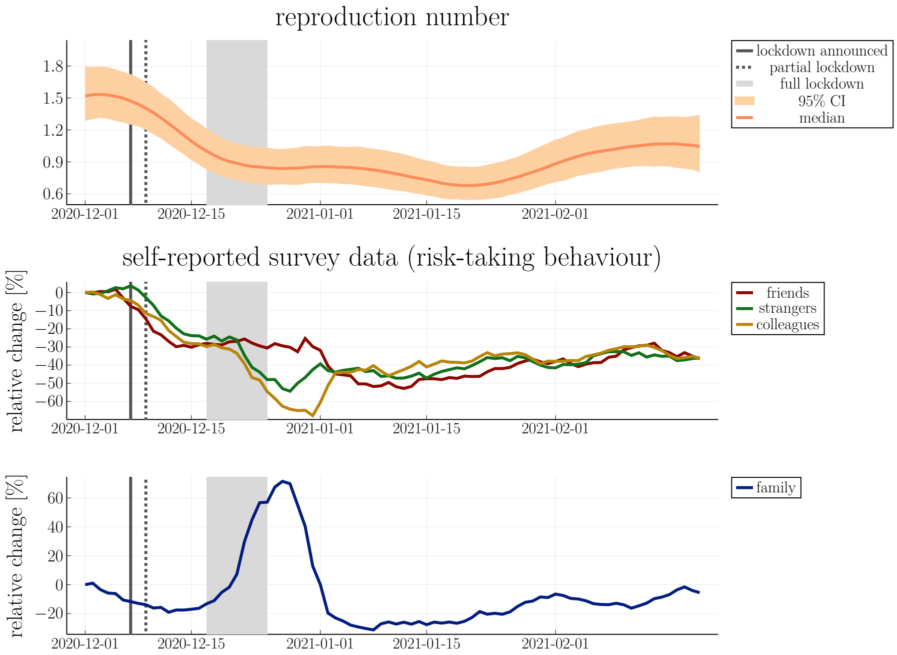

Supplementary Figure 6 Visual national-level comparison between R_t and context-depending risk-taking behaviour. 1st row: Reproduction number R_t inferred from national hospitalizations. 2nd row: risk-taking behaviour towards friends, strangers, and colleagues with a threshold at the 70th percentile. 3rd row: risk-taking behaviour towards family members.

rank	predictor	score difference	score difference (std)
**0**	**friends**	**-0.0**	**0.0**
**1**	**strangers**	**-11.9905**	**7.51123**
2	colleagues	-77.1731	11.5445
3	family	-120.87	12.5731

Supplementary Table 5 Risk-taking behaviour towards friends and strangers predict the observed hospitalizations best and colleagues performs only marginally worse. In detail, we define risk-taking behaviour with a threshold at the 70th percentile, calculate the PSIS-LOO score, which approximates the out-of-sample predictive performance, and rank the results from highest to lowest performing. We consider the score difference significant if it is larger than the 95% CI (approx. twice the standard error) and highlight rows with bold letters that show a non-significant performance difference to the best performing model. Here, risk-taking behaviour towards colleagues and family members outside the household perform significantly worse. However, this observation does not imply that the respective contacts are irrelevant for disease transmission. A joint model that includes all four predictors reveils that contacts to colleagues and family members have highly correlated effect sizes (see Supplementary Fig. 1), suggesting that a combination of both data streams. Indeed, we find that risk-taking behaviour towards colleagues and family members together have a similar predictive performance to the best model (see Supplementary Table 1).

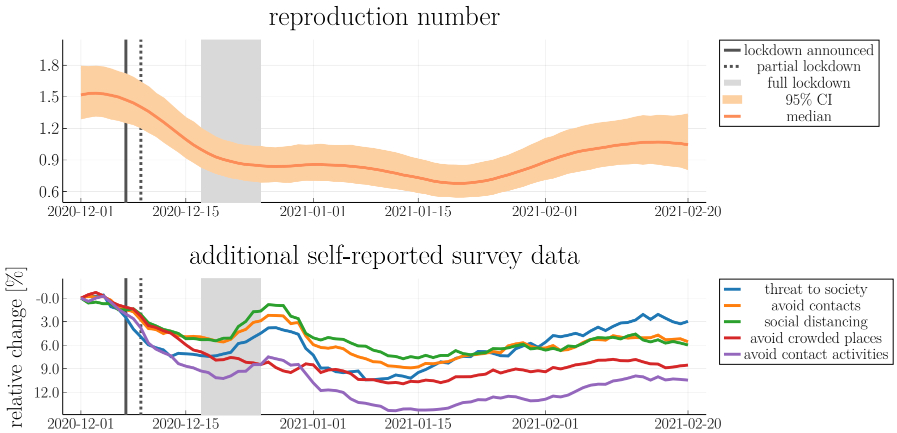

Supplementary Figure 7 Comparison betweeen national-level R_t and additional behavioural time-series from the HOPE survey in the upper and lower panel, respectively. The latter represent changes in the mean response to a number of additional survey questions. The dynamics reflects a similar, though inverted, patterns to R_t and risk-taking behaviour (see Supplementary Fig. 4). Therefore, these time-series support our argument that our survey captures early behavioural changes around the lockdown's announcement. The details: Participants responded on a 1-to-7 scale from ”not at all” / ”completely disagree” to ”to a high degree” / ”completely agree”. The labels in the second panel correspond to the following questions / statements from the survey: (1) The Corona virus is a threat to Danish society. (2) To what extent did you yesterday avoid contacts? (3) To what extent did you yesterday keep 1-2 meters distance to other people? (4) To what extent did you yesterday avoid going to crowded places? (5) To what extent did you yesterday minimize activities where you have contact to other people? We took a seven-day moving average of the mean response value. Supplementary Table 6 evaluates the predictive performance of the above time-series in terms of PSIS-LOO scores.

rank	predictor	score difference	score difference (std)
**0**	**risk-taking behaviour**	**-0.0**	**0.0**
1	avoid contact activities	-9.21759	3.53417
2	avoid crowded places	-19.835	6.09498
3	avoid contacts	-33.1312	6.88432
4	social distancing	-47.014	8.52191
5	threat to society	-122.325	11.8105

Supplementary Table 6 Predictive performance for risk-taking behaviour and additional behavioural time-series from our survey. In detail, we compare risk-taking behaviour given the total number of contacts and a threshold at the 70th percentile against the mean response to additional survey questions. The latter are presented in Supplementary Fig. 7 with details about the questions in the corresponding caption. We calculate the PSIS-LOO score, which approximates the out-of-sample predictive performance and rank the results from highest to lowest performing. We consider the score difference significant if it is larger than the 95% CI (approx. twice the standard error) and highlight rows with bold letters that show a non-significant performance difference. The PSIS-LOO score demonstrates that risk-taking behaviour outperforms indirect measures of behaviour from our questionnaire responses. In addition, this result confirms that our the survey captures early behavioural changes in different aspects of daily life with impact on disease transmission.

date	action
2020-12-07	partial lockdown announced*: significant tightening of Covid-19 restrictions in 38 municipalities across Denmark, including the country's three largest cities, Copenhagen, Aarhus, and Odense.
2020-12-09	partial lockdown in effect
2020-12-16	full lockdown announced**. Nation-wide Restrictions are gradually increased, starting from 2020-12-17 until the full lockdown on 2020-12-25
2020-12-17	shopping malls closed
2020-12-21	school closure & shut down of businesses involving close contact such as
2020-12-24	private events over Christmas are encouraged not to exceed 10 people
2020-12-25	all non-essential retail businesses closed / "full lockdown"

Supplementary Table 7 The above timeline of Denmarks second Covid-19 lockdown follows online media announcements:
* https://www.thelocal.dk/20201207/latest-denmark-announces-partial-covid-19-lockdown-until-2021/, *accessed: 2022-04-20*
** https://www.thelocal.dk/20201216/new-denmark-announces-national-lockdown-from-christmas-day/, *accessed: 2022-04-20.*

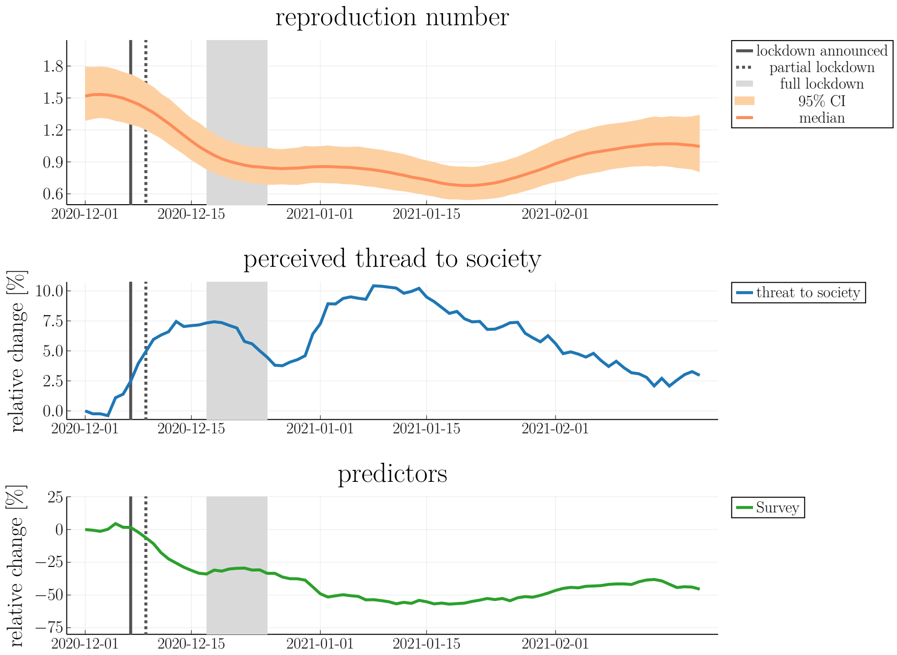

Supplementary Figure 8 Perceived threat of a Covid-19 infection may lead to behaviour change. 1st panel: R_t derived from national-level hospitalizations. 2nd panel: mean response to the statement: *The Corona virus is a threat to Danish society.* 3rd panel: risk-taking behaviour with a threshold at the 70th percentile. The visual comparison suggests that the perceived threat the Covid pandemic leads to a behavioural change as measured by *risk-taking behaviour*.

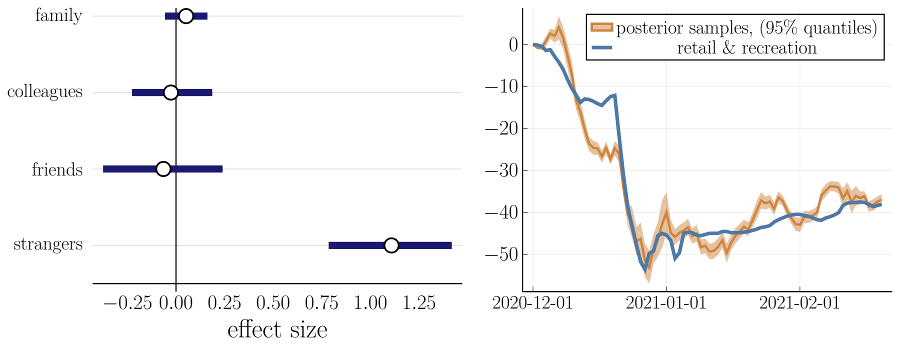

Supplementary Figure 9 Risk-taking behaviour towards strangers explains most of the variation in Google's *retail & recreation* data stream. The details: We fit a linear model with *retail & recreation* as response variable y_t at time t and risk-taking behaviour as covariate X_t^c, where c refers to family, colleagues, friends, or strangers, respectively:

$$
\begin{aligned}
y_t &\sim \mathrm{Normal}(\bar{y}_t, s) \\
\bar{y}_t &= \sum_c e_c X_t^c \\
e_c &\sim \mathrm{Normal}(0, 1) \\
s &= \mathrm{Gamma}(\mathrm{mean} = 5, \mathrm{SD} = 3)
\end{aligned}
$$

We use uninformative prior for the effect sizes e_c and the observation noise s. The left panel shows posterior effect sizes with a circle and bar indicating mean and 95% CI, respectively. The right panel compares the response variable y_t against the generated quantity $\bar{y}_t$ for a visual comparison of the fitting accuracy. We find that contacts to strangers is the dominant predictor for *retail & recreation* and the resulting fit appears in good agreement with the latter.

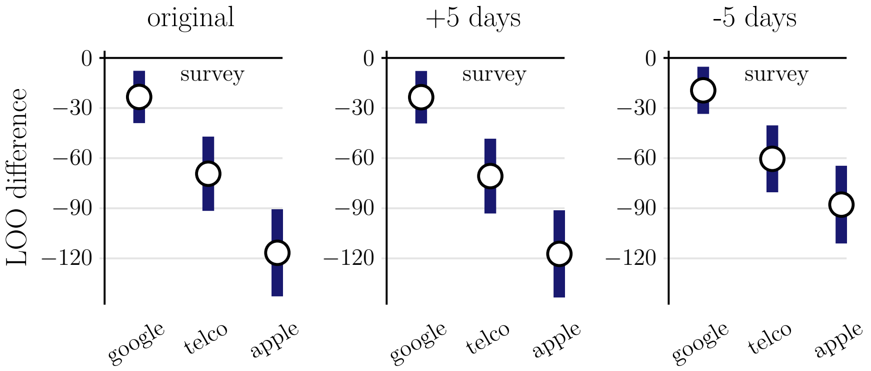

Supplementary Figure 10 The LOO cross-validation results in Fig. 3 of the main text are not sensitive to minor variations in the observation window - Self reported survey is the best predictor for regional hospitalizations compared to the mobility data streams. Details: We compare the result from the main text (left panel) with a shifted observation window: The start and end date are shifted by +5 days and −5 days in the central and right panel, respectively.

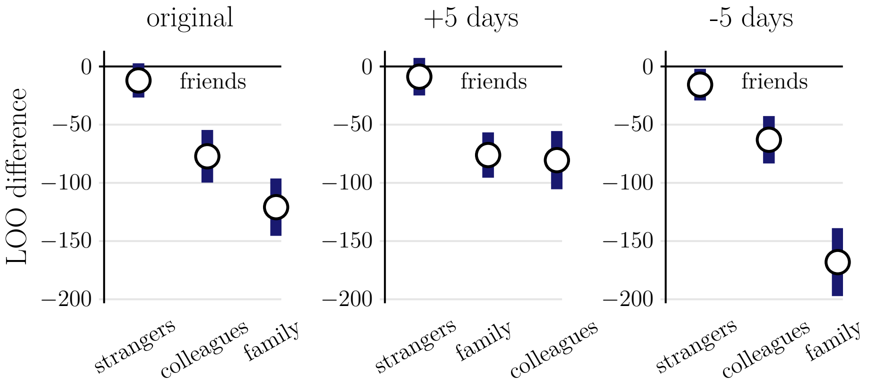

Supplementary Figure 11 The LOO cross-validation results in Fig. 5 of the main text are not sensitive to minor variations in the observation window - Risk-taking behaviour towards friends demonstrates consistantly the best predictive performance and risk-taking behaviour towards strangers performs only marginally worse. Details: We compare the result from the main text (left panel) with a shifted observation window: The start and end date are shifted by +5 days and −5 days in the central and right panel, respectively.

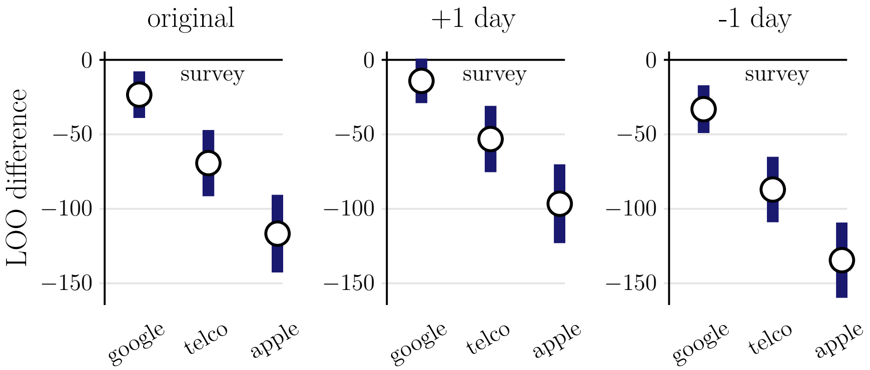

Supplementary Figure 12 The LOO cross-validation results in Fig. 3 of the main text are not sensitive to minor variations in the infection-to-hospitalization delay distribution - Self reported survey is the best predictor for regional hospitalizations compared to the mobility data streams, though *Google* performs only marginally worse in the middle panel. Details: We compare the result from the main text (left panel) with a modified infection-to-hospitalization delay distribution, where we shift the mean of the distribution (Weibull(shape=0.845, scale= 5.506); see Eq. 6) by +1 day (i.e., Weibull(shape=0.845, scale= 6.506)) and −1 day (i.e., Weibull(shape=0.845, scale= 4.506)) in the middle and right panel, respectively. Thereby we keep the shape parameter of the distribution constant.

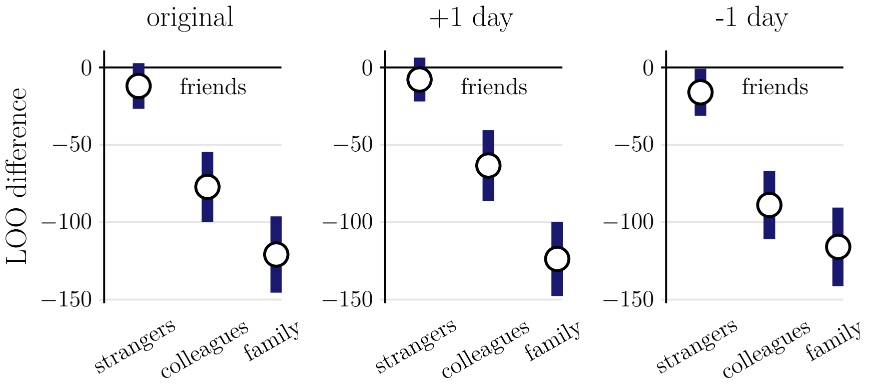

Supplementary Figure 13 The LOO cross-validation results in Fig. 5 of the main text are not sensitive to minor variations in the infection-to-hospitalization delay distribution - Risk-taking behaviour towards friends demonstrates consistantly the best predictive performance and risk-taking behaviour towards strangers performs only marginally worse. Details: We compare the result from the main text (left panel) with a modified infection-to-hospitalization delay distribution, where we shift the mean of the distribution (Weibull(shape=0.845, scale= 5.506); see Eq. 6) by +1 day (i.e., Weibull(shape=0.845, scale= 6.506)) and −1 day (i.e., Weibull(shape=0.845, scale= 4.506)) in the middle and right panel, respectively. Thereby we keep the shape parameter of the distribution constant.

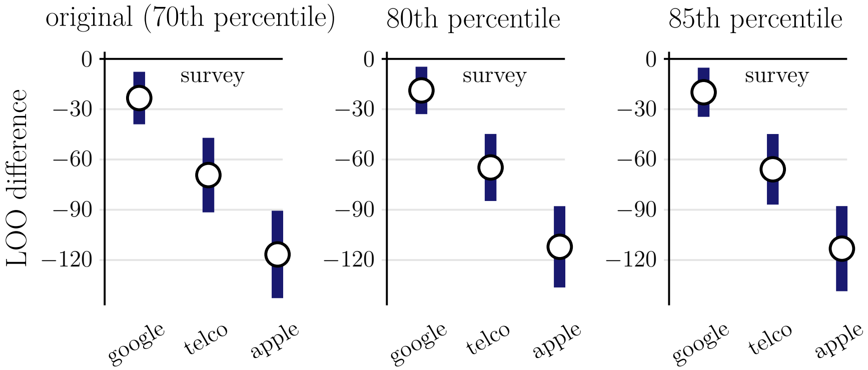

Supplementary Figure 14 The LOO cross-validation results in Fig. 3 of the main text are not sensitive to minor variations in the threshold that defines risk-taking behaviour - Self reported survey is the best predictor for regional hospitalizations compared to the mobility data streams. Details: We compare the result from the main text (left panel) with different choices for the threshold that defines risk-taking behaviour. We choose the 80th and 85th percentile in the middle and right panel, respectively. The thresholds correspond to at least 10, 15 and 19 reported contacts within the past 24h for the 70th, 80th and 85th percentile, respectively.

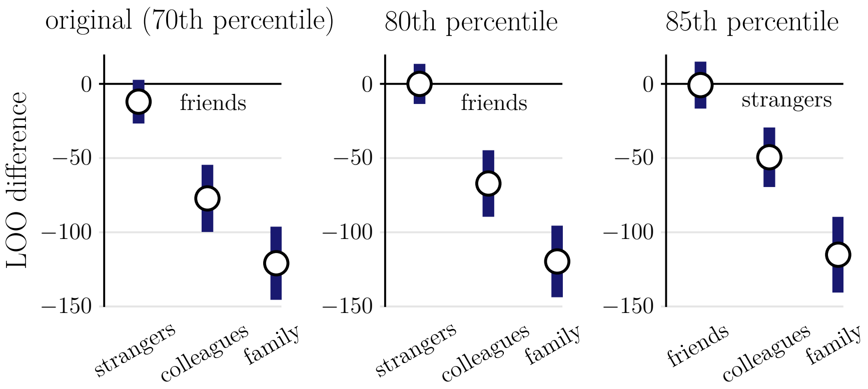

Supplementary Figure 15 The LOO cross-validation results in Fig. 5 of the main text are not sensitive to minor variations in the threshold that defines risk-taking behaviour - Risk-taking behaviour towards friends and strangers demonstrate the best predictive performance. Details: We compare the result from the main text (left panel) with different choices for the threshold that defines risk-taking behaviour. We choose the 80th and 85th percentile in the middle and right panel, respectively. The thresholds correspond to the following number of contacts towards strangers, family members, friends and colleagues, respectively: **70th percentile:** ≥ 1, ≥ 1, ≥ 1, ≥ 1. **80th percentile:** ≥ 4, ≥ 2, ≥ 3, ≥ 3. **85th percentile:**≥ 5, ≥ 3, ≥ 4, ≥ 4

References

[1] Jørgensen, F., Lindholt, M.F., Bor, A., Petersen, M.B.: Does face mask use elicit risk-compensation? quasi-experimental evidence from denmark during the sars-cov-2 pandemic. European Journal of Public Health (2020)

[2] Adam, D.C., Wu, P., Wong, J.Y., Lau, E.H., Tsang, T.K., Cauchemez, S., Leung, G.M., Cowling, B.J.: Clustering and superspreading potential of sars-cov-2 infections in hong kong. Nature Medicine **26**(11), 1714–1719 (2020)

[3] Nouvellet, P., Bhatia, S., Cori, A., Ainslie, K.E., Baguelin, M., Bhatt, S., Boonyasiri, A., Brazeau, N.F., Cattarino, L., Cooper, L.V., *et al.*: Reduction in mobility and covid-19 transmission. Nature communications **12**(1), 1–9 (2021)

[4] Edsberg Møllgaard, P., Lehmann, S., Alessandretti, L.: Understanding components of mobility during the covid-19 pandemic. Philosophical Transactions of the Royal Society A **380**(2214), 20210118 (2022)

[5] Telco mobility data. https://covid19.compute.dtu.dk/visualizations/telco_brush/. Accessed: 2022-03-31

[6] Bezanson, J., Edelman, A., Karpinski, S., Shah, V.B.: Julia: A fresh approach to numerical computing. SIAM review **59**(1), 65–98 (2017)

[7] Ge, H., Xu, K., Ghahramani, Z.: Turing: a language for flexible probabilistic inference. In: International Conference on Artificial Intelligence and Statistics, AISTATS 2018, 9-11 April 2018, Playa Blanca, Lanzarote, Canary Islands, Spain, pp. 1682–1690 (2018). http://proceedings.mlr.press/v84/ge18b.html

[8] Hoffman, M.D., Gelman, A., *et al.*: The no-u-turn sampler: adaptively setting path lengths in hamiltonian monte carlo. J. Mach. Learn. Res. **15**(1), 1593–1623 (2014)

[9] Publicly available source code. https://github.com/andreaskoher/Covid19Survey. Accessed: 2022-05-21

[10] Vehtari, A., Gelman, A., Gabry, J.: Practical bayesian model evaluation using leave-one-out cross-validation and waic. Statistics and Computing (2016). https://doi.org/10.1007/s11222-016-9696-4

[11] Google. Community mobility reports. https://www.google.com/covid19/mobility/index.html?hl=en. Accessed: 2022-03-31

[12] Apple. Mobility Trends. https://www.apple.com/covid19/mobility. Accessed: 2022-03-31
